# Supplementary figures and images for: Landscape of essential growth and fluconazole-resistance genes in the human fungal pathogen Cryptococcus neoformans
Source: PLoS Biol. 2025 May 22;23(5):e3003184. doi: 10.1371/journal.pbio.3003184 (PMC12136443; doi:10.1371/journal.pbio.3003184)

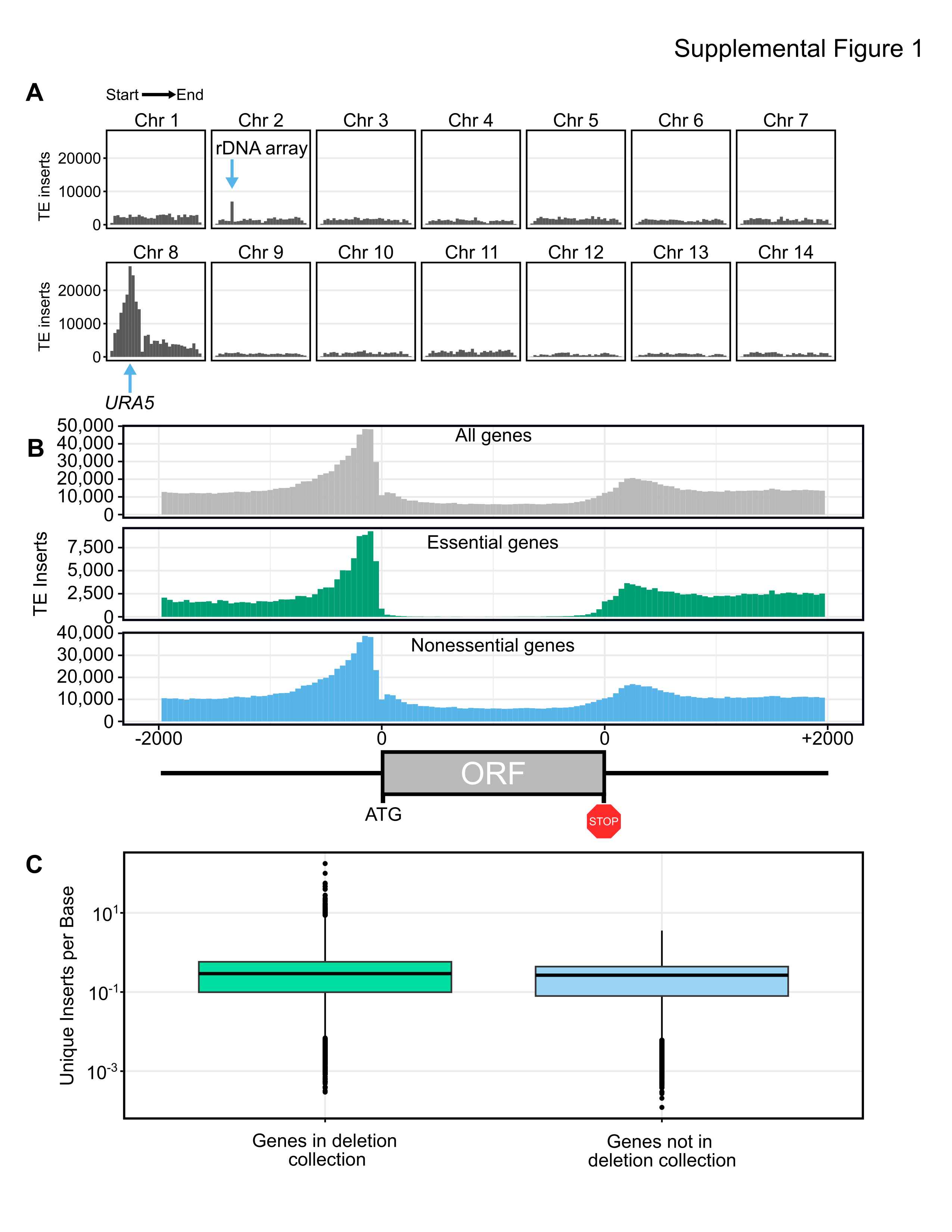

Supplement: S1 Fig — (A) Transposon insertion count is plotted across chromosomes. There are clear peaks at the rDNA array on Chromosome 2 and the URA5 locus on Chromosome 8, as well as the surrounding chromosome. (B) Transposon insertion count is plotted across the 2000 bases immediately upstream and downstream of the start codon. Inserts within the gene bodies are distributed among equally sized windows for each gene. Rows are split based on predicted gene essentiality. (C) Insertions per base are plotted for genes not present versus present in the deletion collection. Boxplots show first quartile, median, third quartile. The whiskers show the range to a maximum of 1.5 times the interquartile range above and below the first and third quartile, respectively. Outliers are displayed as individual datapoints. Data underlying A and B can be found in S1 Data at 10.5281/zenodo.15264486. Data underlying C can be found in S1 Data at 10.5281/zenodo.15264486 and in S1 Table. (TIF) [file pbio.3003184.s001.tif]

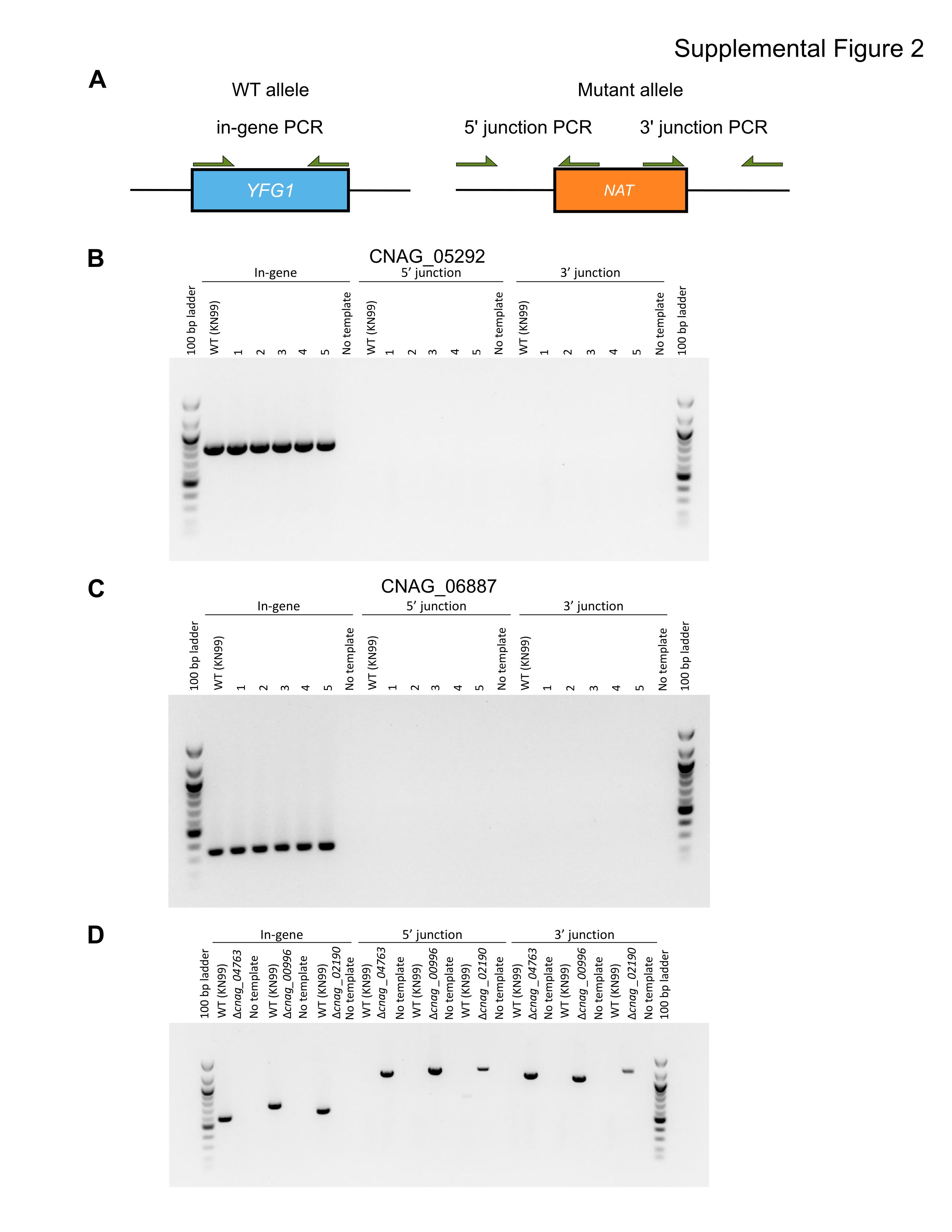

Supplement: S2 Fig — (A) Schematic of PCR validation strategy for deletions. WT alleles should be detectable with an in-gene PCR that amplifies wildtype DNA sequence (left). Mutant alleles should have the wildtype allele replaced with a drug resistance marker. The junctions between the genomic sequence and the resistance marker should be detectable via PCR on both the 5′ and 3′ ends (right). A successful mutant should produce bands from the 5′ junction and 3′ junction but not from the in-gene PCR. A wildtype strain should produce a band only from the in-gene PCR. (B) PCR validations for 5 independent colonies from the deletion collection strain for CNAG_05292. All five produce wildtype in-gene bands and no junction bands. (C) PCR validations for 5 independent colonies from the deletion collection strain for CNAG_06887. All five produce wildtype in-gene bands and no junction bands. (D) PCR validations for deletion collection strains for CNAG_04763, CNAG_00996, CNAG_02190. All three strains produce negative in-gene PCRs and successful junction PCRs for both 5′ and 3′ ends. Original gel images can be found at 10.5281/zenodo.15264486 as S1 Raw images. (TIF) [file pbio.3003184.s002.tif]

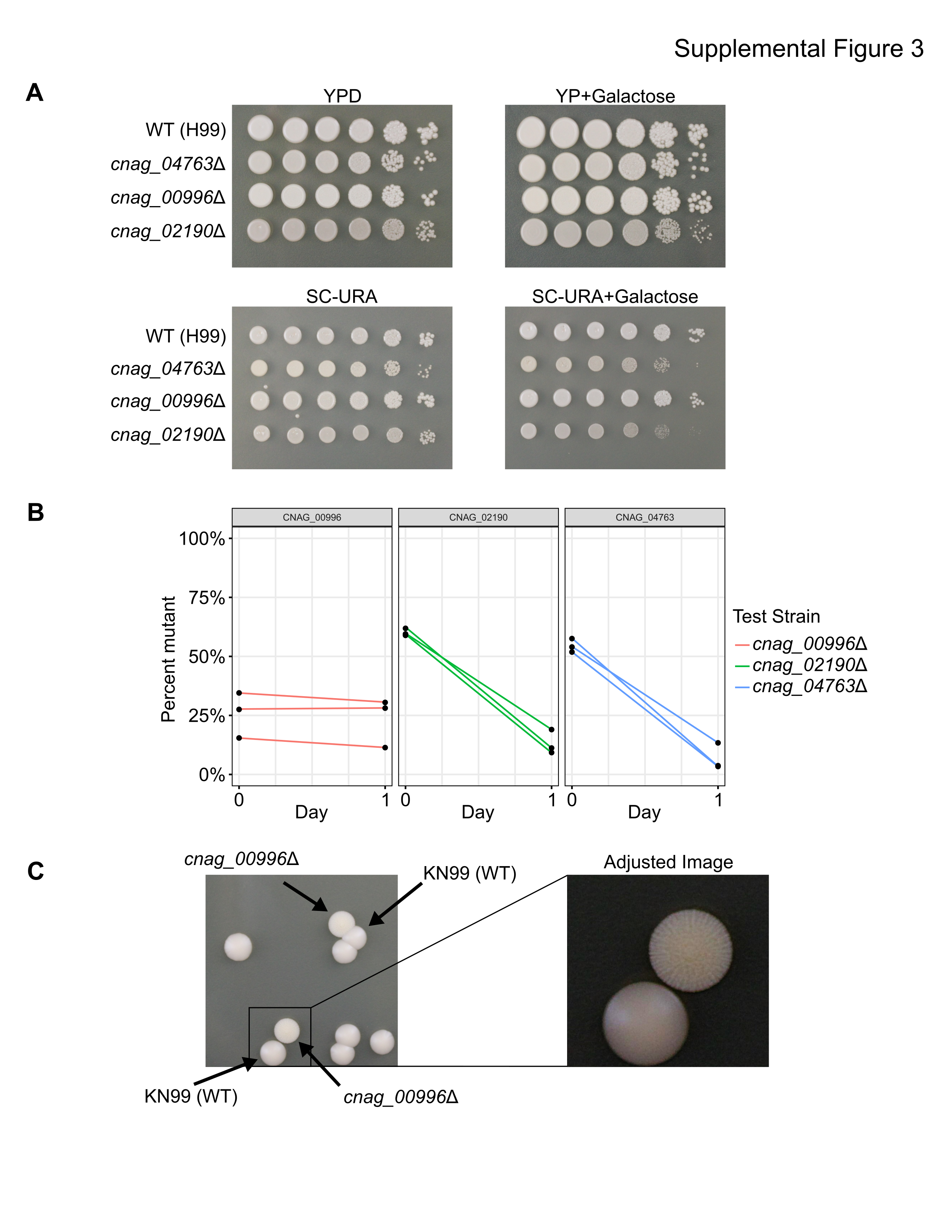

Supplement: S3 Fig — (A) Spot dilution assays with 5 μL spots plated. The initial leftmost spot is of OD600 = 20 culture and each successive spot is a 10-fold dilution, so that the final spot should be 105 less concentrated than the first. All four plates were spotted on the same day with the same dilution series. (B) Competition assay with percentage of mutant plotted on the y-axis. Each mutant was competed against the same unmarked wildtype KN99 parental strain. Strains were competed in the SC-URA + Gal media used in the original assay. Strains were originally mixed at a 50:50 ratio based on OD600. Inconsistency with mixing of CNAG_00996 suggests an altered OD600 to CFU ratio. (C) Picture of colonies on YPD plates from the competition assay for the cnag_00996Δ mutant. Colonies were distinctly different in appearance and replica plating to YPD + NAT media confirmed that rough colonies were the mutant strain. Panel on right is zoomed in from inset box on left and is adjusted to help visualize difference between colonies more clearly. Original images underlying A and C can be found in the Stowers Original Data Repository at http://www.stowers.org/research/publications/libpb-2480. Data underlying B can be found at 10.5281/zenodo.15264486 as S3B Fig. (TIF) [file pbio.3003184.s003.tif]

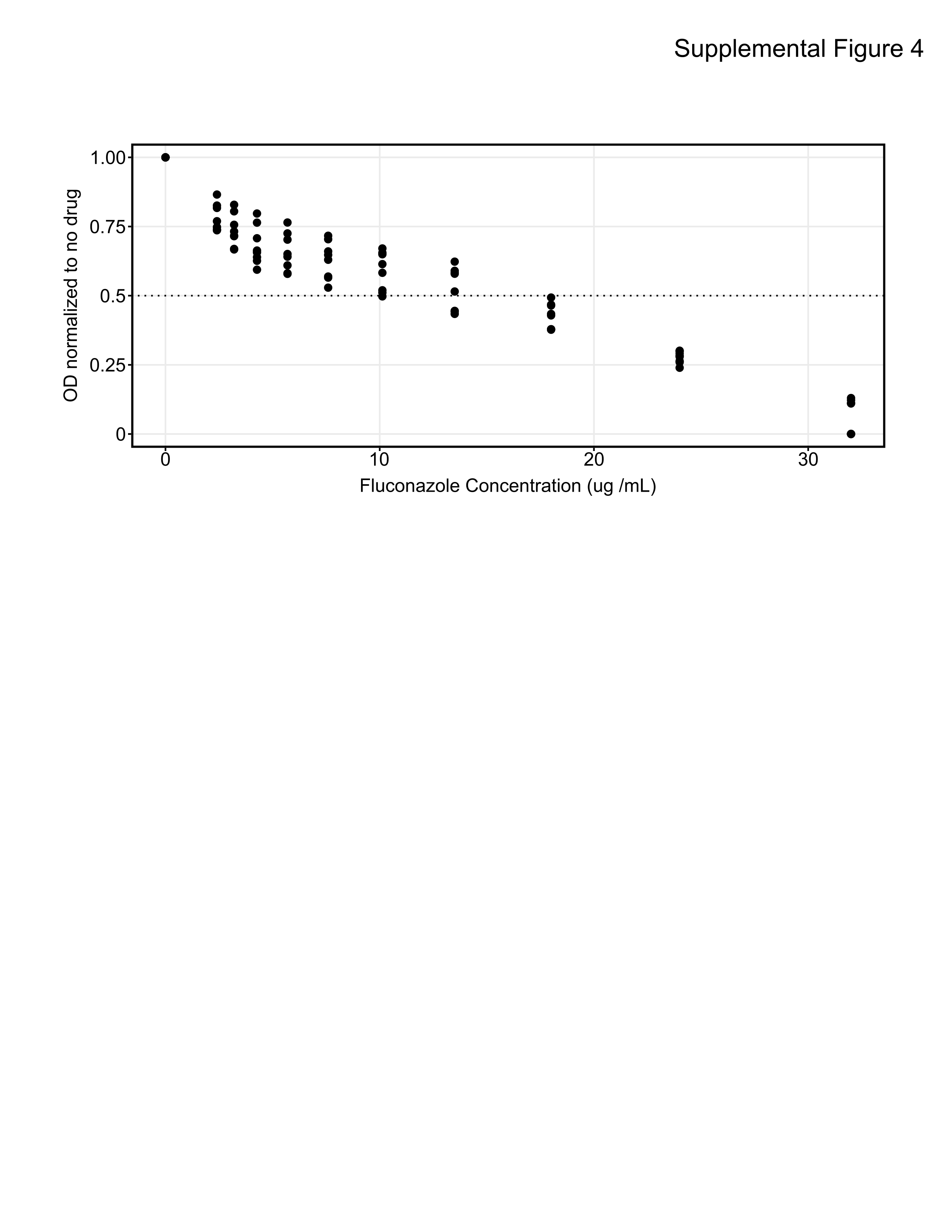

Supplement: S4 Fig — Growth at 24 h plotted relative to a no drug control. X axis displays concentration of fluconazole added in DMSO. Y axis shows OD600 normalized to OD600 of no drug control. The underlying data can be found at 10.5281/zenodo.15264486 as S4 Fig. (TIF) [file pbio.3003184.s004.tif]

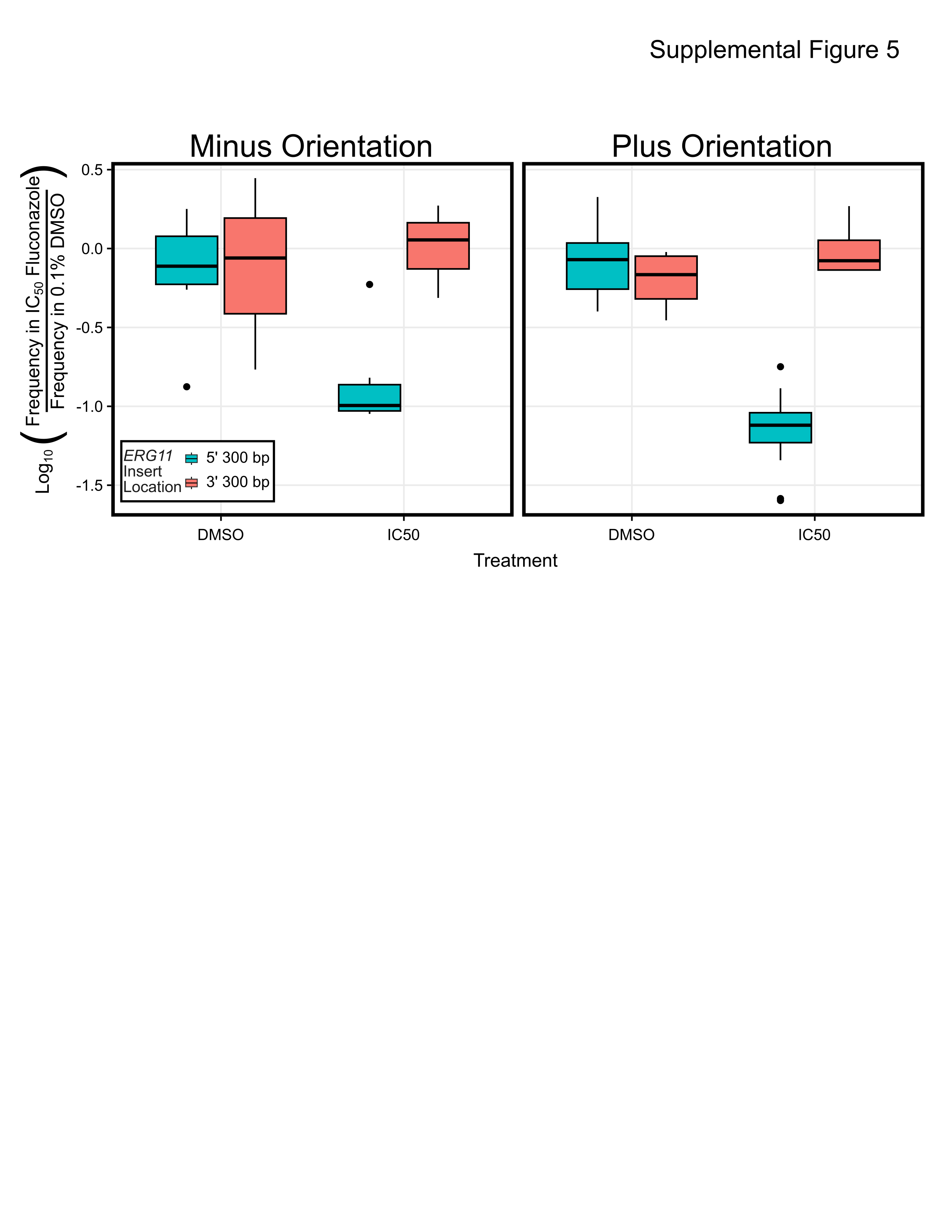

Supplement: S5 Fig — Boxplot displaying distribution of log10-adjusted fold changes in insert density (i.e., frequency in fluconazole/frequency in DMSO). Each column shows inserts only within a 300 base pair region either immediately upstream of the start codon or downstream of the stop codon. Boxplots show first quartile, median, third quartile. The whiskers show the range to a maximum of 1.5 times the interquartile range above and below the first and third quartile, respectively. Outliers are displayed as individual datapoints. Inserts are split based on whether they are oriented in the same direction as ERG11 (plus) or the opposite orientation (minus). Underlying data can be found in S1 Data at 10.5281/zenodo.15264486. (TIF) [file pbio.3003184.s005.tif]

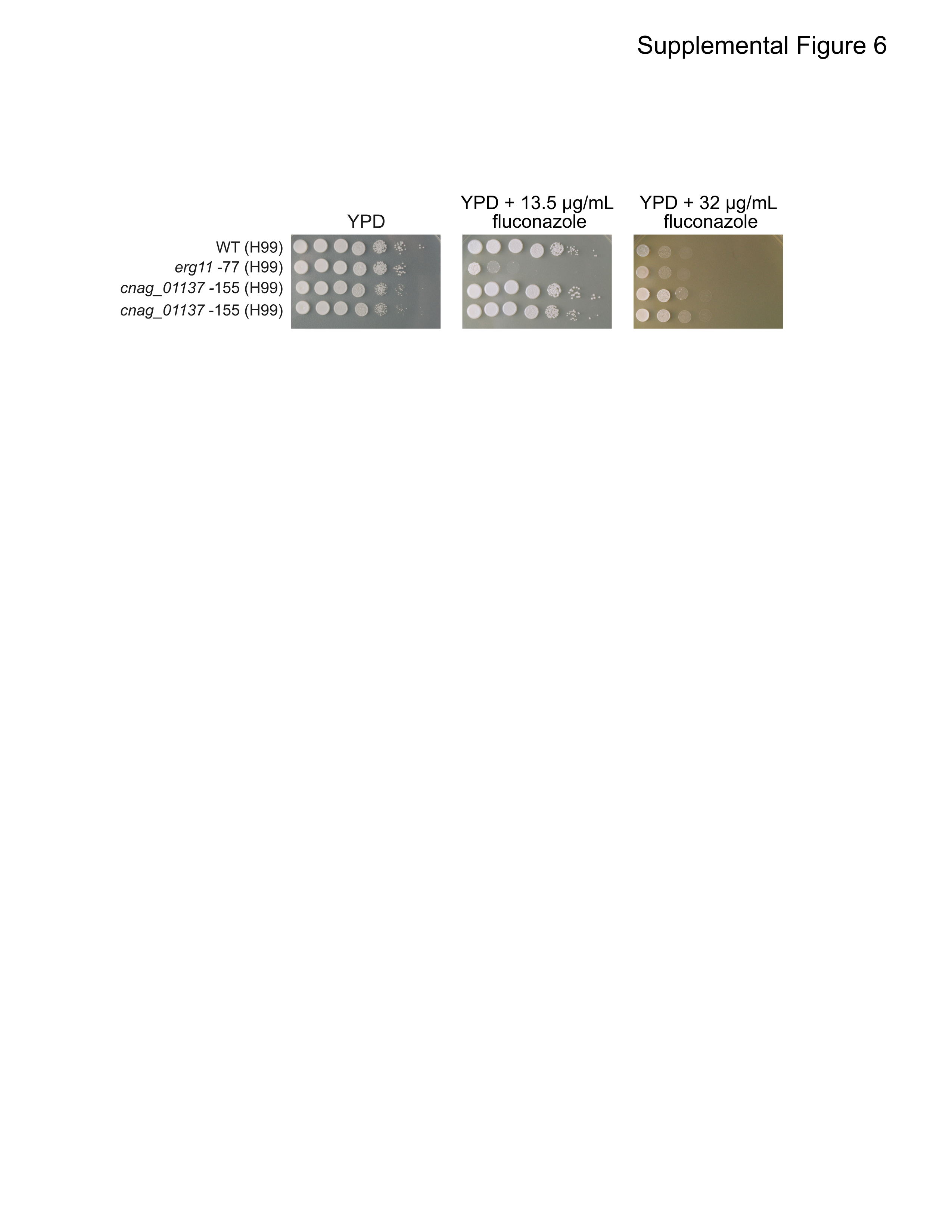

Supplement: S6 Fig — Spot dilution assays with 5 μL spots plated. The initial leftmost spot is of OD600 = 20 culture and each successive spot is a 10-fold dilution, so that the final spot should be 106 less concentrated than the first. All plates were spotted on the same day with the same dilution series. YPD plates were imaged after 48 h at 30°C and fluconazole plates were imaged after 72 h at 30°C. Original images can be found in the Stowers Original Data Repository at http://www.stowers.org/research/publications/libpb-2480. (TIF) [file pbio.3003184.s006.tif]

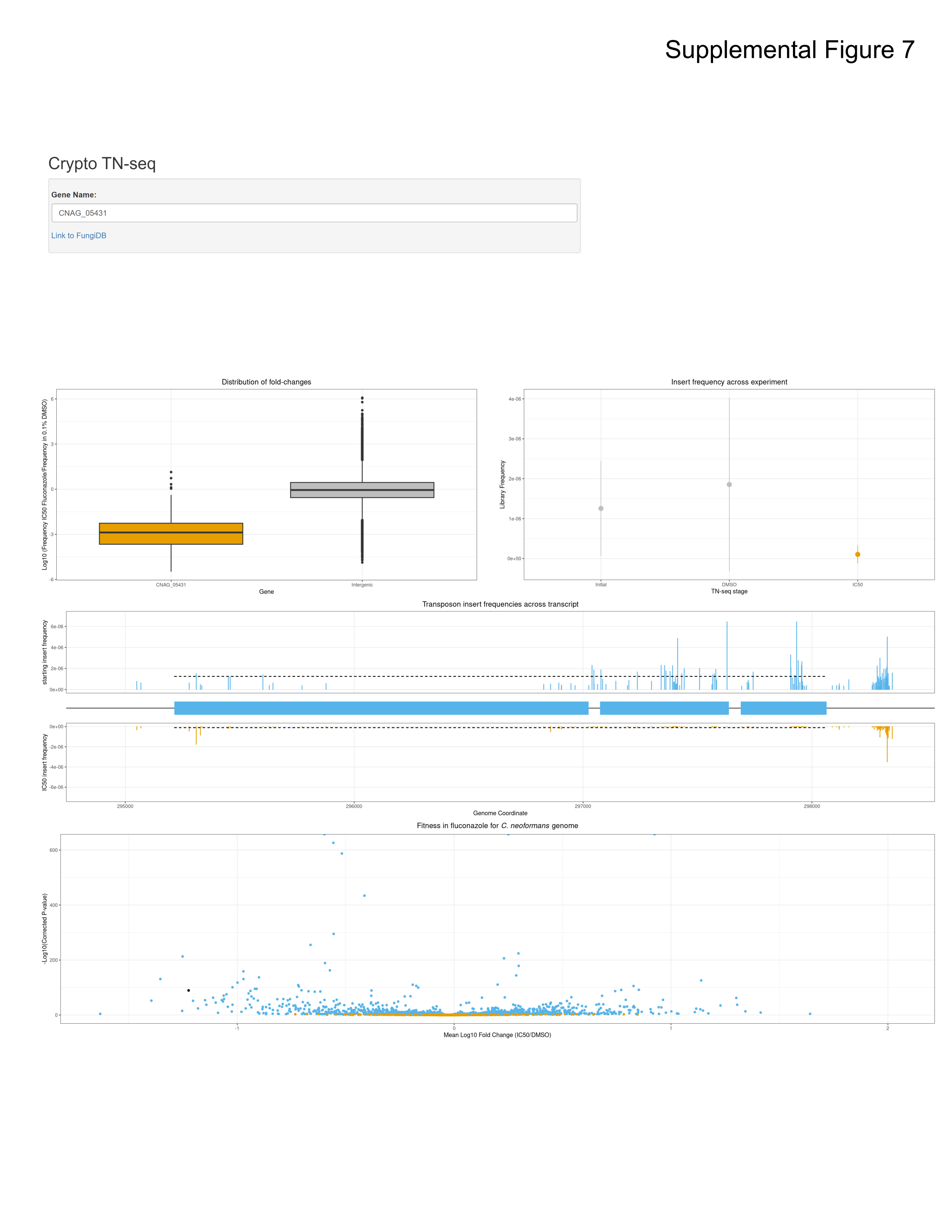

Supplement: S7 Fig — Screenshot of a publicly available interactive Shiny app (https://bbillmyre.shinyapps.io/Crypto_TN_seq_viewer/) that visualizes data from the C. neoformans TN-seq assay. There are four plots, displaying the distribution of fold changes within a gene compared with intergenic inserts (as in Fig 4C,) the distribution of insert frequencies across a gene at three different experimental stages, transposon insertion frequencies across a gene with an additional 300 bases before the ATG and after the stop codon, and finally a volcano plot (as in Fig 4B) with the current gene highlighted in black. The app only accepts C. neoformans systematic names (i.e., CNAG_0####). Underlying data can be found in S1 Table and in S1 Data at 10.5281/zenodo.15264486. (TIF) [file pbio.3003184.s007.tif]

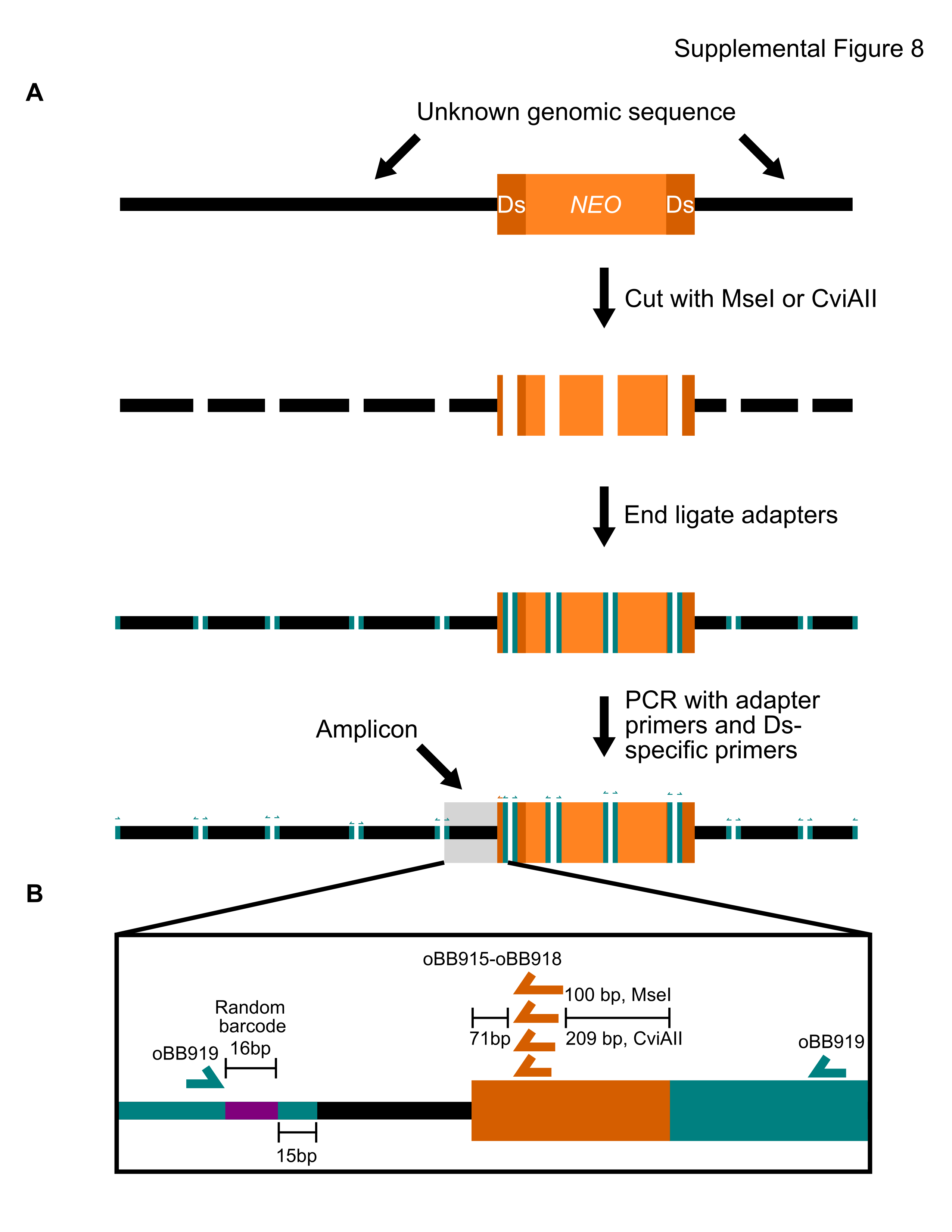

Supplement: S8 Fig — (A) Diagram showing sequential digests and adapter ligation/PCR to amplify the boundary between the known Ds sequence and the unknown genomic sequence. Notably, primers cannot amplify between the two ligated adapters because the ligated adapters are modified so that they can only serve as the template in a second-round reaction. (B) Zoom in of primers amplifying the transposon insertion. The four Ds specific primers anneal to the same location but have varying lengths so that the products produced will have a diversity of starting nucleotides when sequenced. (TIF) [file pbio.3003184.s008.tif]

B

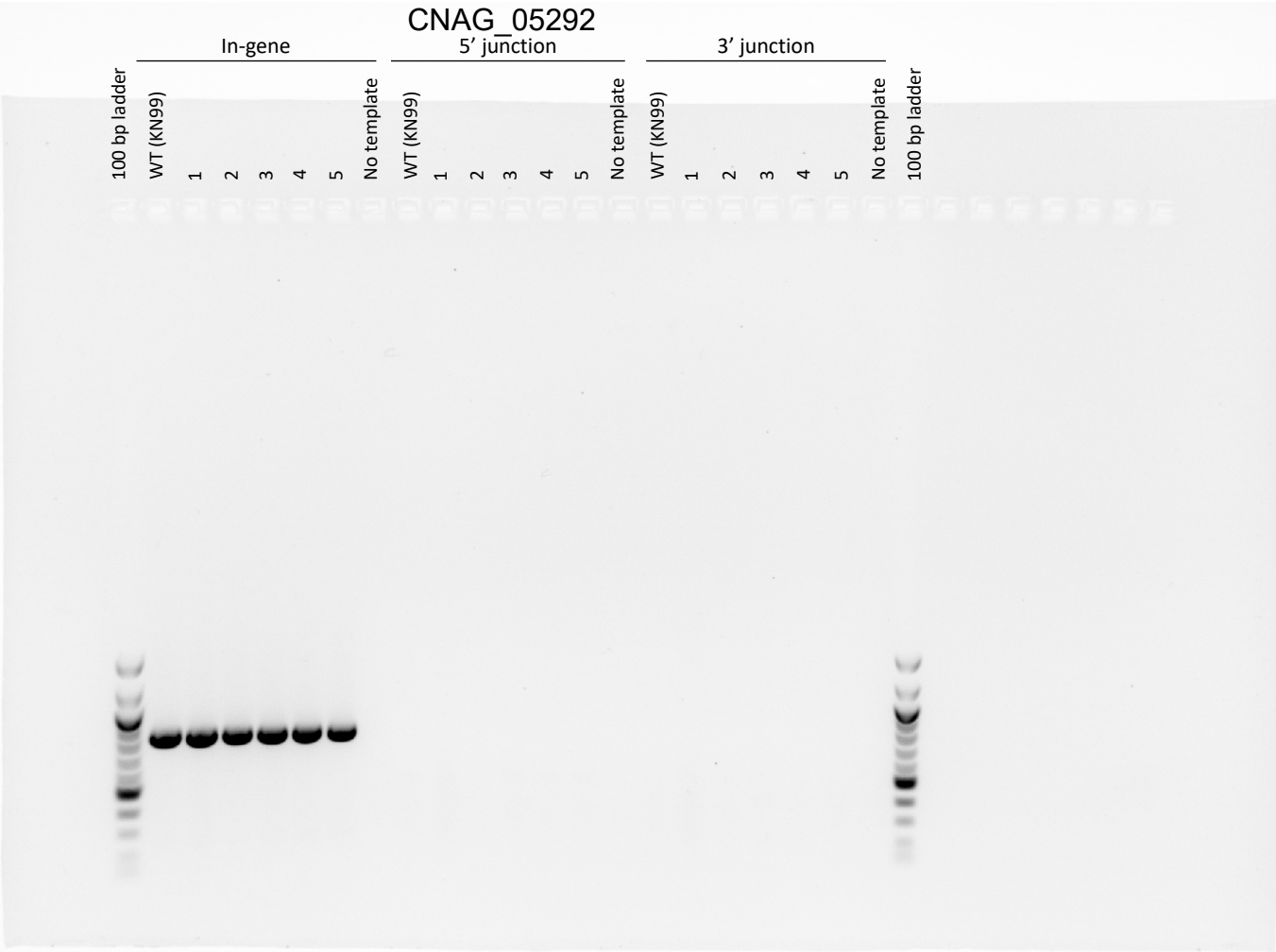

C

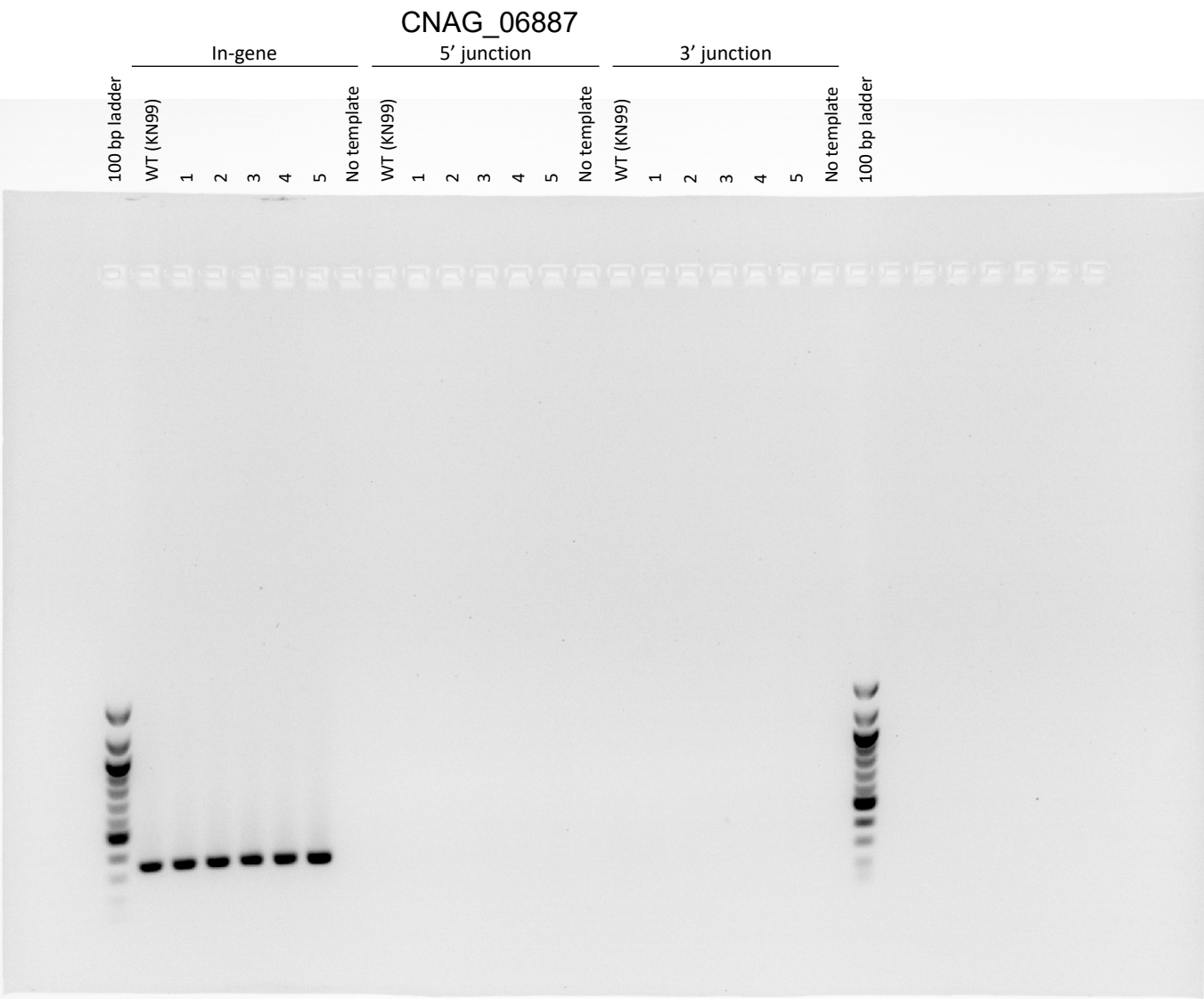

D

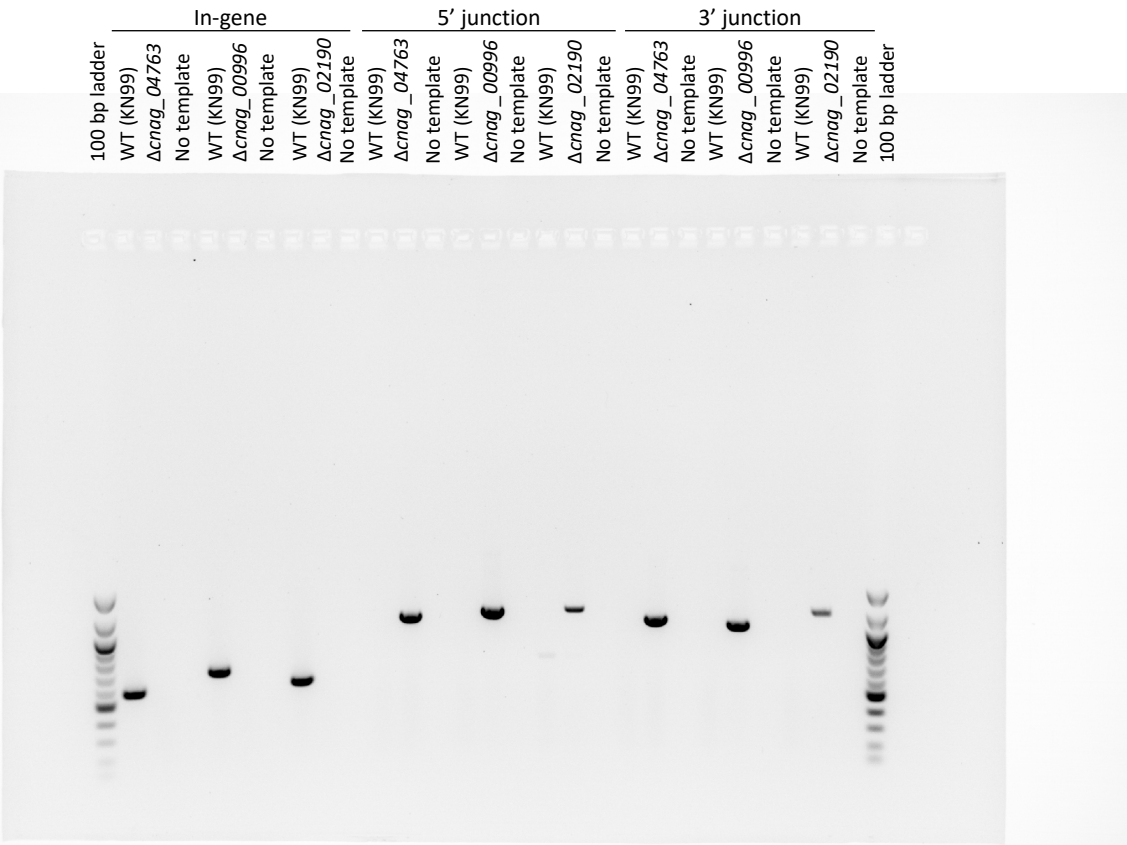

Supplement: S1 Image — (PDF) [file pbio.3003184.s016.pdf]
