# Supplementary material for: Landscape of essential growth and fluconazole-resistance genes in the human fungal pathogen Cryptococcus neoformans
Source: PLoS Biol. 2025 May 22;23(5):e3003184. doi: 10.1371/journal.pbio.3003184 (PMC12136443; doi:10.1371/journal.pbio.3003184)
Supplement: Attachment — Submitted filename: Response to reviewers.pdf [file pbio.3003184.s019.pdf]

Editor:

In light of the reviewer reports included below, we would like to invite you to revise your manuscript to address the feedback provided. Reviewer #2, in particular, raised concerns about the study's broad interest. Reviewer #1 requests testing cps25 on fluconazole plates and examining whether mitochondrial genes affect other antifungals. Additionally, Reviewer #2 suggests deleting specific mitochondrial genes to further support their role in fluconazole resistance, and requests a metagene plot to visualize the density of insertions in intergenic regions. We agree with these suggestions, which will require some additional experimental work, as addressing these concerns will significantly strengthen the manuscript. We also want to emphasize the importance of testing additional antifungals to broaden the study's relevance.

Reviewer #1:

Cryptococcus is a critical priority human fungal pathogen (as designated by the WHO) and is very much understudied. It is rapidly becoming the model basidiomycete. This study provides a very important dataset, which will be extremely useful to the Cryptococcus community (of the essential genes including those genes that can only tolerate transposon insertion in their 5' UTR). The shiny app makes this dataset very accessible: I have tried this out for my 'favourite genes' and am very impressed with the data described.

The study also provides an excellent example of the power and the many uses that can be made of the TN-seq technique. Overall the findings are novel and of significance to the Cryptococcus community and well beyond - for those interested in drug resistance, and those keen to try out this powerful technique in their own system. I am not an expert on machine learning and big data analysis, but the statistical analysis seems to me to be appropriate and I believe that all data needed to replicate the study are being made available here. The supplementary information will be very valuable to the Cryptococcus community.

The manuscript is well written and the data presented very clearly in the figures. As such, I am very supportive of the publication of this manuscript. Below, I suggest a few changes to be made before publication.

We thank the reviewer for their very positive comments and agree that we expect this to be a resource for the community. We have had several groups reach out either to ask questions about specific genes or to thank us for the Shiny app. The paper was uploaded to *bioRxiv* at the same time it was submitted to *PLOS Biology* and that preprint is already accumulating citations as well, indicating the general interest in the field.

Figure 4: on page 28 it is pointed out that several components of the Rim1 pathway were identified amongst the hits for fluconazole sensitivity. The authors should briefly explain here what is known about the functions of this pathway (rather than having the reader wait for this information, which is provided in the discussion). Why was vps25 not tested on the fluconazole plate? This should be included for completion.

As suggested, we have added a brief description of the functions of the *RIM101* pathway to the results following its first mention.

VPS25 was not tested on the plates simply because the Alspaugh Lab (where we acquired our RIM pathway mutants) did not have a *vps25*Δ mutant in their collection at the time. However, our dataset clearly indicated this was nonessential. We checked the Madhani deletion collection and there was a *vps25*Δ mutant in the collection that we were able to PCR validate and include in a new version of the spot dilution assays that is now included in the paper as a new version of figure 4D. As expected, this mutant is also highly sensitive to fluconazole. Thank you to the reviewer and apologies for this oversight on our part!

Figure 5: this is an important figure demonstrating a very useful facet of TN-seq mutagenesis. Many mitochondrial genes were identified here, suggesting that perturbed 'general' mitochondrial functions can lead to fluconazole resistance. However, I doubt this is specific to fluconazole resistance. The authors should test whether these strains are 'multi-drug resistant'. I think it likely that they have several stress response pathways activated and are possibly overexpressing several efflux pumps etc. The authors can refer to reviews and specific papers on
this. eg. https://doi.org/10.3390/microorganisms8101574; www.pnas.org/cgi/doi/10.1073/pnas.1911560116; doi:10.1128/EC.05184-11 (review).

This is an interesting suggestion from the reviewer. We decided to test this hypothesis with a few different strains, including two newly constructed 5' insertions upstream of an essential mitochondrial gene (CNAG_01137, aconitate hydratase) and four deletions of nonessential mitochondrial genes found in the Madhani deletion collection (CNAG_00768 (unnamed, ETC complex I associated protein), CNAG_03375 (*LSC1*), CNAG_04064 (*MIX17*), CNAG_04212 (*MIC19*)). With the exception of a mild increase in resistance to 5FC in one of the two 5' inserts upstream of CNAG_01137, none of the mutants had changes in resistance to 5FC or Amphotericin, while all showed increases in resistance to fluconazole, albeit mild in the case of the CNAG_01137 mutants. We have included this data in a new panel (Figure 5F) and new supplemental figure (Supplemental Figure 6). We have also added additional commentary about the hypothesis suggested by the reviewer as well as one alternate model. Blocks in mitochondrial metabolism that slow the TCA cycle will lead to less acetyl-CoA entering the TCA cycle. Acetyl-CoA is also the first step in ergosterol synthesis, which is inhibited by fluconazole. We hypothesize that the slowdown in mitochondrial metabolism may shunt acetyl-CoA to ergosterol synthesis, partially overcoming Erg11 inhibition.

Reviewer #2:

TN-seq in Cryptococcus neoformans

Billmyre et al

The authors describe a comprehensive application of TN-Seq with Cryptococcus using machine learning to identify essential genes. They apply this method to identify genes involved in fluconazole resistance. In characterizing intergenic insertions, they find that the density of insertions upstream of genes can be used to characterize the contributions of essential genes that otherwise cannot tolerate insertions in their ORFs.

This is a carefully conducted set of experiments combined with a scholarly description of the method. The quality of writing is high and the results well documented. And for the most part the interpretations are solidly supported by the data. The result is a valuable list of essential genes in Cryptococcus and a list of genes involved in fluconazole resistance. This data provides an important resource for future work on drug resistance. Although I find nothing of significance is wrong with this work, I must say that TN-Seq has been used on fungi for over 10 years including experiments to determine genes involved in fluconazole resistance. My view is this is a quality study but that it may not be of wide interest to the readers of PLOS Biology. As this is a study of quality genetics, I think it will be of high interest to readers of important genetic journals.

We greatly appreciate this very thorough review from Henry Levin. Henry's expertise in transposons and TN-seq has enabled us to produce what we believe is an improved manuscript in revision. Respectfully, we disagree that this will not be of wide interest to the readers of *PLOS Biology*. The data and tools generated here are of very broad use to the Cryptococcus community. We have already provided prepublication access to the data from this publication to multiple labs, many of which will cite that data in upcoming manuscripts. We've provided support letters for grants applications using these tools to multiple additional labs, one of which has already successfully received funding.

Further, while model yeasts are an incredible resource, studies of drug resistance are most valuable when performed in the specific organism. *Cryptococcus neoformans* is a basidiomycete and diverged from the ancestors of the ascomycetes approximately 600 million years ago. This means that there has been substantial opportunity for divergence in the pathways mediating drug activity and resistance. We previously found a novel drug resistance mechanism to the antifungal drug 5FC in *C. neoformans* that was missed because the relevant genes had been lost in the Ascomycetes, including *S. cerevisiae* and *C. albicans*, but maintained in *Cryptococcus* (Billmyre et al. 2020, PMID: 31913284). We do not believe exploring fluconazole resistance in *Cryptococcus* is redundant nor without broad interest.

Specific comments

* Please use page numbers and or line numbers. Its hard to refer to specific text without them. I will use the page numbers of the PDF as listed by Adobe Acrobat.

Apologies! I often find myself writing this comment as a reviewer as well and am unsure why I failed to include them. Page and line numbers make this much easier for everyone and we have added them in the revised version. We've done our best to line up the reviewer's comments with the manuscript and address specific comments appropriately.

* Abstract, line 11, We show that 5' insertions… I would specify insertions 5' of genes.

Added.

* Page 13, 7 lines from bottom. Please provide reference support for statement about transmission.

Added.

* Page 14, line 8. Please provide the total number of genes in Cryptococcus.

Added.

* Page 14, 10 lines from bottom. There are at least two papers demonstrating deletion sets have a high frequence of calling essential genes nonessential. Deletion strains carry aneuploids and rearrangements. Guo et al 2013, and PMID: 10888885.

Added.

* Page 14, 6 lines from bottom. There are other references of TN-seq to include. Cerevisiae study of prion pathology PMID: 29769283, Galbrata PMID: 32819971, Yarrowia PMID: 29792930, Albicans study with Azole resistance PMID: 30374049.

Added.

* Page 14, second line from bottom. Should comment that TN-seq requires haploid genomes.

Added.

* Page 15, line 2. there are a variety of computational approaches. You may want to compare the advantages of machine learning. A list of computational approaches is provided in PMID: 32886545.

We have added this citation and briefly discussed motivation for choosing machine learning in the results section.

* Page 15, line 8. The limitations of CRISPR include lack of information about essential genes. And regions/genes not annotated correctly cannot be tested. TN-Seq can identify intergenic and noncoding sequences that have function.

We completely agree with the reviewer. We have added more details about annotation to this section in the discussion.

* Page 15, bottom. 3rd line from bottom. Not the first use of TN-seq to query essential gene function. The structure of this sentence confuses how all TN-seq studies identify essential genes and how some can query specific function of essential genes. Lee et al measured contributions of essential genes to heterochromatin function. And Mitchel et al studied individual

domains in essential proteins by TN-seq. Best to modify your claim to focus on upstream insertions.

We appreciate this comment. We drew this distinction between our 5’ upstream inserts and the previous reports based on the assumption that some essential genes are more likely to be capable of tolerating inserts in subdomains (as in Michel) or in 3’ regions (as in Lee) than other genes. This means that approaches that look at coding inserts may find some essential genes with a role in a given function but likely not all of them. However, while we suspect that 5’ upstream inserts may be more generalizable, 5’ upstream inserts also likely have some similar issues, as a subset of essential genes are likely unable to tolerate expression perturbation of any kind. We have thus revised this section to clarify as suggested:

> “To our knowledge, this is the first use of TN-seq to query essential gene function via perturbation of 5’ regulatory regions in fungi.”

* Page 16, line 8. Typo, unlikely capable of splicing…

Fixed.

* Page 17, line 6. This statement describes selection for excision not transposition. G418 is used to select for insertions. I would mention that here. Also, I would like to see more detail in your methods regarding the size of the random barcode and the the PCR primers to know the distance between the PCR primer sequence and the random barcodes. perhaps a drawing in the supplement would help.

Fixed. Incidentally, while in early trials we did double select with SC-URA and G418 to select both for excision and transposition, we eventually realized that the vast majority of excision events resulted in transposition in our hands even without G418 selection. The final libraries used here were G418 selected post transposition out of an abundance of caution, but we suspect it may be unnecessary in *Cryptococcus neoformans*.

We have added a cartoon schematic of the library preparation process as Supplemental Figure 8, including the sizes of random barcodes and distances between primer sites.

* Page 17, 4 lines from bottom. Local hoping is a very interesting behavior that has been observed for many TEs including Hermes, Ds, sleeping beauty, piggybac. P etc.

We have added a reference to local hopping.

* Page 17, Suppl. Fig 1B. This is a very accurate description of insertions across the start codon. But it would also be very interesting to see a metagene plot across genes. Hermes in pombe and cerevisiae showed higher level of integration on either side of the ORF due to nucleosome distribution.

We appreciate this suggestion and have changed Supplemental Figure 1B to a metagene plot. Essentially, instead of centering insert data on the start codon, we've centered the data on genes and normalize all genes to the same length so that insert frequency can be visualized on either side of the gene. We think this improves on our original data presentation. Like past observations, we also saw increases in TE integration on either side of the ORF with preference for the immediate upstream region.

* Page 18, line 5. is this kb of the entire genome or of the mappable genome minus repeat sequences, rDNA.

These numbers are per kb of the entire genome.

* Machine learning section. I would like to commend the authors for their scholarly description of the machine learning methods. This builds confidence in the predictions. I would suggest that their first failed approach that trained with the 89 unknown genes be moved to the supplement.

Thank you for the suggestion. We have revised this section to shorten the description of the first model and moved the relevant details from the removed sections to the methods.

* Page 18, 9 lines from bottom. In addition to ref 36, PMID: 32886545 contains a (at the time) comprehensive list of TN-seq experiments including in eukaryotes.

Citation added.

* Page 19, 3rd line from bottom. Remind the reader the 1,653 genes are the conserved set.

Fixed.

* Page 20, 8 lines from bottom. I would find better language than unknown genes. Its their essentiality that is unknown.

We appreciate this suggestion and have replaced "unknown" with "unclassified."

* All Supplemental Tables. They really need legends that describe their content. Can be in a separate page of the excel files.

We have added legends in a new sheet for supplemental tables 1-3 to explain the column headers. Supplemental tables 4-6 are Strain, Plasmid, and Primer tables and headers are more self-explanatory.

* Page 22, last line. including the absence of an in-gene PCR product.

Added.

* Page 23, 10 lines from bottom. Did you find genes required to induce the Gal promoter. Wouldn't these be misclassified as essential? Discuss here?

We would expect to find genes required for growth on galactose (including both the transcriptional activators and metabolic genes) misclassified as essential. We looked at homologs of several *S. cerevisiae GAL* genes in *Cryptococcus neoformans*. Two homologs of *GAL10* are both essential in our data, while the *GAL7* ortholog is not confidently scored by our model. *GAL1* has two homologs in *C. neoformans* called *GAL1* and *GAL101,* neither of which our model scores as essential. Perhaps there are redundant functions in this setting or perhaps Gal1p has slow turnover and these mutants are capable of persisting until cells were expanded in glucose containing YPD. We have added a brief discussion at line 299.

* Page 24, 11 lines from bottom. This is a valuable list for future study. Will the authors discuss a subset of best to target.

Added.

* Page 25, 1st line. These 34 genes are of great value and should be listed.

Agreed. We have added a new supplemental table to highlight these genes. In the process, we have also added a further filter for those lacking human orthologs. The orthomcl approach we used from FungiDB included a set of genes as lacking human orthologs that had particularly high divergence but likely still have orthologs and are identified as such by SGD (eg. Cse4 and CENP-A). Because highly divergent genes are probably still very relevant for drug design, we now report both the direct FungiDB numbers (n=34) and the further SGD filtered numbers (n=17).

* Page 26, line 2. Are these genes particularly small causing large gaps per length of gene.

No, the average length of this set of 50 genes (2048.98 bases) is slightly longer than the average gene (1903.37 bases). We have added to the text in the results.

* Page 26, 8 lines from top. If genes are duplicated or have very similar sequences, then insertion reads cannot be uniquely mapped giving the appearance of being essential.

We thank the reviewer for this comment. We were specifically worried about this potential issue and chose to err on the side of incorrectly calling more genes nonessential rather than risk erroneously calling repetitive genes essential. To do so, we mapped repetitive reads promiscuously. By default, the aligner we used (BWA mem) allows multiple alignments and reports mapping scores of 0 in case of multi-mapped reads. This means that we can map inserts within a repetitive gene, we simply can’t tell which copy of that repetitive gene they originated within. In cases of truly identical gene sequences, we would detect identical insert landscapes for those two copies. Multi-mapped reads are relatively rare and represent less than 5% of the alignments produced. We have added text in the methods to make this approach more clear.

* Page 28, Fig. 4B, purple color of the RIM pathway is hard to see.

We have changed to a different shade of purple to increase contrast.

* Page 28, 8 lines from bottom. Would be helpful here to have a comment defining the RIM pathway.

Both reviewers suggested this and we have added a brief description of the functions of the *RIM101* pathway to the results following its first mention.

* Page 29, line 6. hypomorphs of essential genes can also be generated by c-terminal insertions in coding sequence. Do you see this? Also, this is transposon dependent. Some TEs have promoters that drive expression of adjacent genes (Michel-2016).

We have not formally tested this model. We definitely see individual essential genes that tolerate inserts at the 3 or 5 prime end but unlike 5' transcription, this was far from universal and we found it challenging to define a specific window that would allow us to effectively conduct a statistical test across all essential genes. Potentially we could do so by manually defining windows for the 1,465 essential genes defined here, but that work is not trivial and so we have utilized a more generalizable route with the 5' insertions. We have discussed the TE/promoter issue in an answer below.

* Page 29, 10 lines from bottom. But it appears that insertions within the ERG11 ORF were tolerated in fluconazole. Or at least ERG11 was not placed in Fig. 4B or C. It is possible you didnt have the statistical power in the ORF. With Hermes there is much higher density of insertion in the intergenic sequences. Perhaps that is true for Ac/Ds. A metagene plot would reveal this.

ERG11 was not placed in either Figure 4B or 4C because there were too few coding inserts to have statistical power because *ERG11* is a known essential gene (as displayed in Figure 2A). This can also be seen in the new figure 5A, which has a zoom in of the region surrounding *ERG11*.

* Page 29, 7 lines from bottom. A map of the insertions upstream of ERG11 would provide visual confidence.

We have introduced a new version of Figure 5 in part to address this. New panel 5A includes a zoom in of the insertions upstream of *ERG11*.

* Page 29, 3 lines from bottom. Is there orientation specificity to the insertions in the upstream sequence. I believe there is a cryptic promoter on one end of your Ac/Ds.

Thank you for this comment. The SATAY system in *S. cerevisiae* previously identified effects from a cryptic promoter for *S. cerevisiae* within the dS transposon. We checked this

originally because we anticipated directional effects of transposon inserts such that inserts in either the + or – orientation would have gain of function effects. However, we found no real effect of insert orientation for *ERG11*. We have included a version of this figure in the revised manuscript (below, new Supplemental Figure 5). Unlike Michel et al, we also did not see enrichment of TE inserts in the promoter regions of genes with known loss of function defects in fluconazole. For example, *AFR1* encodes a known drug efflux pump that acts on fluconazole. We saw loss of TE inserts not only in the coding region of *AFR1*, but also in the upstream region. We suspect this difference may be caused by differences in the drug marker encoded within the transposon. The standard *S. cerevisiae* drug markers are known to utilize bidirectional promoters (PMID: 36503721). We have added a note and citation about this possible explanation in the results. Alternately it could simply be a result of the evolutionary distance between *C. neoformans* and *S. cerevisiae*, which is estimated at approximately 600 million years. What is a cryptic promoter in *S. cerevisiae* does not appear to be operating as one in *C. neoformans*.

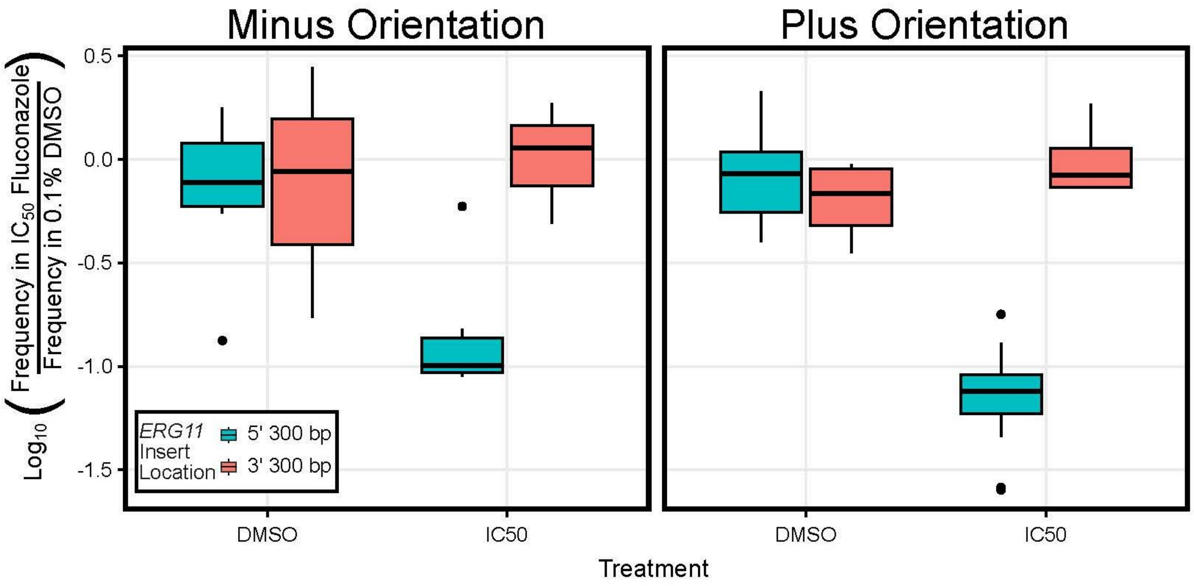

*	Page 30, 8 lines from bottom. Your insertions could also be increasing expression of the mitochondrial genes. Mitchel et al found Ac/Ds has a cryptic promoter capable of expressing essential domains. To support the loss of mitochondrial function model of fluconazole resistance I would test a few deletions of mito genes?

See comments above about cryptic promoters. In addition, to address comments from reviewer 1 above, we have identified several nonessential mitochondrial genes and constructed and tested one 5' insertion strain as well, all of which display phenotypes on fluconazole consistent with a loss of function model of fluconazole resistance. This data is now included as Figure 5F.

*	Page 30, 3 lines from bottom. This is an excellent use of TN-Seq maps. It would be best to illustrate this point by showing a map of the ERG11 insertions with and without drug.

This is now included as figure 5A.

* Page 31, Shiny app. This app is a great addition. I did have trouble looking up genes with three letter names. Does it only recognize systematic names? I would suggest a page of instructions. I was very interested in looking at the insertions around ERG11 but because insertions in the ORF are low its not in the app. I suggest you show the insertion patterns regardless of the statistical significance of the ORF. This will allow people to look at the promoter regions.

We appreciate the comments. The three letter names are difficult for *Cryptococcus neoformans* because many genes still lack established names and the community lacks a well curated resource like SGD or Pombase that would disambiguate names that have been used multiple times. Adding three letter names would really be a community level curation process and there has not been sufficient community interest or effort committed to this problem. Instead, we implemented the systematic names here. We do include insert patterns around genes that lack sufficient inserts but omit the first two plots that are displaying coding inserts specifically.

* Page 33, 10 lines from bottom. Typo, Here we find…

Fixed.

* Page 36, top line. The reader should be reminded that TN-Seq only works with haploids.

Added.
